# Responses of snow leopards, wolves and wild ungulates to livestock grazing in the Zorkul Strictly Protected Area, Tajikistan

**DOI:** 10.1371/journal.pone.0208329

**Published:** 2018-11-30

**Authors:** Khalil Karimov, Shannon M. Kachel, Klaus Hackländer

**Affiliations:** 1 University of Natural Resources and Life Sciences (BOKU), Institute of Wildlife Biology and Game Management, Vienna, Austria; 2 Academy of Sciences of Tajikistan, Institute of Zoology and Parasitology, Dushanbe, Tajikistan; 3 University of Washington’s School of Environmental and Forest Sciences, Seattle, WA, United States of America; 4 Panthera, New York, NY, United States of America; Université de Sherbrooke, CANADA

## Abstract

Long recognized as a threat to wildlife, livestock grazing in protected areas has the potential to undermine conservation goals, via competition, habitat degradation, human-carnivore conflict and disruption of predator-prey relationships. In the Strictly Protected Area Zorkul in Tajikistan (Zorkul Reserve), grazing is commonplace despite official prohibition, with potentially detrimental effects on local fauna, in particular, snow leopard *Panthera uncia*, wolf *Canis lupus*, brown bear *Ursus arctos*, argali sheep *Ovis ammon*, Asiatic ibex *Capra sibirica*, and long-tailed marmot *Marmota caudata*. To understand the impacts of grazing and associated human pastoralism on the large mammal community in Zorkul Reserve we used data from camera traps to build models of ungulate and carnivore site use intensity, and we investigated carnivore summer diets using microscopic scat analysis. While sample sizes limited our inference for several species, we found that site use of the most common ungulate, argali, decreased with proximity to herder’s camps, indicating possible displacement into sub-optimal habitats. However, no such pattern was present in carnivore site use. For wolf and snow leopard, the most frequently encountered prey items were argali and marmot, while bear depended almost exclusively on marmot. While current pastoralist practices in the reserve may not be incompatible with wildlife presence, our findings suggest that pastoralism may negatively impact ungulates by displacing them from otherwise suitable habitats, with unknown fitness consequences for ungulates or the predators that depend upon them. Managing Zorkul Reserve and other actively grazed protected areas to meet potentially competing demands of local pastoralist communities and conservation will require careful consideration of such interactions to minimize the risk of cascading negative impacts on wildlife.

## Introduction

At global and local scales alike, livestock grazing and associated pastoralist activities pose a pervasive yet insufficiently understood threat to native wildlife and ecosystems [1,2]. Livestock can reduce forage availability for native ungulates [2,3], disrupt predator-prey interactions, increase the frequency and intensity of human-carnivore conflict [4,5], and pass on disease to native species [6]. Furthermore, livestock and humans may exclude wild ungulates from otherwise suitable habitat [3,7], and likewise prompt shifts in carnivore space use [8] and temporal activity pattern [4,9], with potentially important fitness consequences and implications for conservation if humans and their livestock prevent wildlife from accessing critical resources.

In Central Asia, policies in designated protected areas that officially prohibit livestock grazing are only sporadically enforced in practice [10], leading to heavily skewed ratios of livestock to native ungulate biomass [11] with potentially divergent, context-dependent implications for wild herbivores [12] and their predators [13]. For example, Namgail et al. [9] observed when livestock arrived at winter pastures used by argali *Ovis ammon*, the argali were forced to shift to marginal habitats with low vegetation cover, although they did not leave the area. Similarly, Fedosenko and Blank [14] suggested that grazing throughout the mountains of Central Asia forces argali into more rugged terrain than they would otherwise use. Bagchi et al. [3] found that livestock competed with Asiatic ibex *Capra sibirica* for forage or displaced them altogether, presumably into marginal habitats. Likewise, Rovero et al. [7] found that livestock displaced ibex, but not necessarily their predators, as their presence did not prompt shifts in snow leopard *Panthera uncia* space use, an observation consistent with previous work showing that snow leopard habitat use was positively associated with livestock densities up to some threshold [13]. In a protected area in Mongolia, where livestock were estimated to account for more than 90% of all herbivore biomass, livestock and associated pastoralism were associated with decreased food availability, potentially increased disease prevalence, and increased predation by domestic (herder) dogs [12]. Furthermore, livestock supplanted argali as the basis of wolf *Canis lupus* diets, and effectively decoupled wolf demographic processes from wild prey abundance. Pastoralists simultaneously subsidized and suppressed wolves (through retaliatory killing), thereby mediating not only apparent competition between livestock and native herbivores, but also apparent facilitation [12], while also subsidizing domestic dogs, which were themselves a major source of mortality to argali [15]. Within protected areas, such human- and livestock-induced disruption of basic ecological interactions among native wildlife may undermine foundational conservation goals.

We sought to assess the potential influence of livestock and associated pastoralist activity on wild ungulates and their predators–wolf and snow leopard–in the Zorkul Reserve of Tajikistan, in order to assess the compatibility of pastoralism with wildlife conservation goals in the eastern Pamirs. Livestock may outnumber native ungulates in the surrounding region by an order of magnitude [16], and despite the protected area designation, Zorkul Reserve is used as summer pasture by local communities [10]. We investigated the relationship between pastoralism and wild ungulate and carnivore habitat use using photo-based occupancy models that considered multiple hypothesis-driven explanatory variables related to terrain features, forage availability, and the proximity of humans and livestock. Additionally, we investigated the importance of livestock in carnivore diets, in order to further understand potential mechanisms underlying the patterns we observed. We hypothesized that ungulate distributions, but not carnivore distributions, would be affected negatively by human and livestock proximity [3,7], and that carnivore diets would include only a small portion of livestock, because their natural prey, mountain ungulates, particularly argali, as well as medium-sized prey like long-tailed marmot *Marmota caudata* [17], were broadly available across the study area.

## Study area

**Fig 1:**
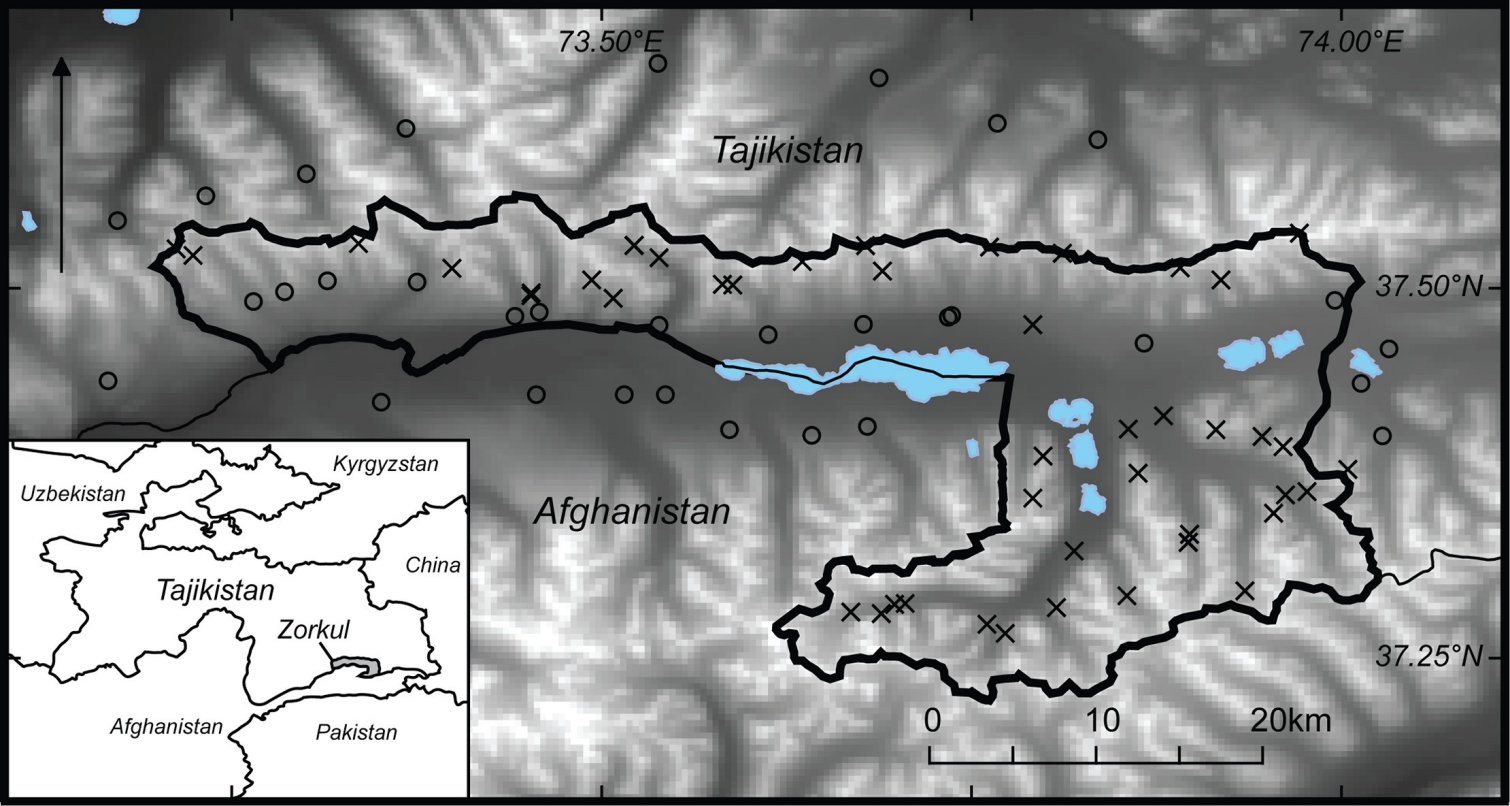
Zorkul Reserve, Tajikistan, 2015 camera trap (crosses) and livestock herder’s camp locations (with dots). Higher altitudes in lighter color and lakes in blue.

The Zorkul Reserve (37.45° N, 73.70° E, Fig 1), established in 2000 [18] with the goal of biodiversity conservation and protection of the unique alpine ecosystem free of any direct influence caused by human economic activities, is an IUCN Category 1A [19] protected area in Tajikistan situated in the Eastern Pamirs on the border with Afghanistan. In practice, local communities have used the alpine wetlands and meadows of the reserve as pasture for livestock for generations, and continue to do so despite the legal designation of the area. Zorkul Reserve is rich in regionally important water resources, notably including extensive glaciers and rivers, which feed large alpine lakes and wetlands that provide habitat for fish and other wildlife species, including migratory birds. Roughly half of the 887 km^2^ area of the reserve consists of valley bottom meadows, wetlands and gentle lower slopes, while the remaining area is characterized by rocky, rugged alpine slopes, ridges, glaciers, and cirques. The flora of Zorkul Reserve is characterized by alpine steppe, desert and meadows. Graminoids such as *Carex spp*., *Elymus spp*., *Kobresia spp*., *Poa spp*., and *Stipa spp*. are dominant on meadows and near wetlands. The alpine meadows are covered by semi-shrubs and forbs, most frequently *Artemisia spp*., *Eurotia ceratoides*, and *Oxytropis spp*. Altitude ranges from approximately 4000 to 5500 m above sea level. The reserve is home to a suite of large carnivores requiring expansive habitat [5], including snow leopard, wolf, and brown bear *Ursus arctos*. Herbivores include argali, Asiatic ibex, long-tailed marmot, hares *Lepus tolai*, and pika *Ochotona macrotis*, all of which are potentially, during at least some portion of their life histories, vulnerable to predation by all three large carnivores [20]. According to reserve staff and herders alike, the reserve is a regionally significant lambing area for argali. In addition, numerous raptors and mesocarnivores, including red fox *Vulpes vulpes*, prey on ungulates, rodents, and lagomorphs in Zorkul Reserve. Various birds, including the bar-headed goose *Anser indicus* (the flagship species of the reserve), and fishes, such as the Pamir false osman *Schizopygopsis stoliczkai* and marinka *Schizopyge curvifrons*, also dwell in the reserve [21].

Seasonal grazing is the dominant human land use in the reserve. Based on digitized maps produced as the result of interviews with herders at each of the active herder’s camps in the reserve in August 2015, we estimated that 285 km^2^ of the lower elevations of the reserve were grazed routinely each year. However, we could not assess the spatial extent of yak grazing given the practice of allowing yaks to range freely and widely. A simple tally of livestock reported suggested that there were more than 4200 sheep, 2700 goats, 1100 cows, 400 yaks, 100 donkeys, and 60 domestic dogs during the period of this study, with herd sizes ranging from 150 to 800 animals. There were 18 active, stationary herder’s camps within the reserve area with corrals to protect livestock during the night. In the 2-year period of 2015 to 2016, during which time livestock numbers were seasonally stable, herders in Zorkul Reserve reported wolf predation on only four domestic animals. On the Afghan side of the international border there were roughly 11000 domestic animals in an area of approximately 400 km^2^, mostly sheep and goats. Based on interviews with herders there, conflict between carnivores and herders during the summer (June through September) is rare, and 90% of all wolf attacks occur in the winter (November through March), when approximately 150 units of livestock are killed annually by wolves (pers. comm., Stepháne Ostrowski, Wildlife Conservation Society).

## Methods

### Camera trapping and site use modeling

We deployed motion-triggered camera traps (Reconyx Hyperfire 800 and Panthera v5) targeting carnivores at 45 sites (Fig 1) throughout the Zorkul Reserve from August 1, 2015 to November 8, 2015. The mean distance between neighboring cameras was 2620 m (± 1497 SD), and the minimum distance was 600 m. We deployed cameras at sites with abundant carnivore signs (e.g., scats, tracks, scent marking sites) or at sites frequented by other wildlife (e.g., trail junctions, ridgelines, passes). Cameras were deployed for a total of 2839 trap nights, though operational performance of individual cameras varied during the study period, with a mean camera site operation period of 63.89 days (± 31.1 SD).

To estimate the influence of natural and anthropogenic factors upon ungulate and carnivore space use, we used species-specific binary detection/non-detection data from camera traps to estimate the probability of site use *ψ* and detection *p* under an occupancy framework in the ‘unmarked’ package [22] in R version 3.3.2 (R Core Development Team 2011). We used single-season occupancy models [23] to investigate the combined impact of human and livestock proximity on the spatial distribution of large mammals, while accounting for imperfect detectability and other possible explanatory environmental variables. Occupancy models adopt a hierarchical model structure to separately model general linear covariate effects on both occurrence, *ψ*, and the conditional process of detection, *p*, given occurrence [22]. Given the mobility and large scale of ungulate and carnivore space use relative to our camera array, our approach was to estimate not occurrence or occupancy per se, but rather intensity of site use [22,23]. This approach estimated intensity of use as a constant, while allowing site-specific probability of detection to vary throughout the sampling period.

We grouped our detection data into 10 sampling occasions of 10 days each, resulting in a site by occasion binary detection matrix for each species. This 10-day sampling occasion length was chosen to reduce zero-inflation arising with low daily probability of detection, while minimizing information loss associated with reducing multiple within-occasion detections to a single binary detection/non-detection variable. We derived a set of potential explanatory covariates for consideration in candidate models in QGIS (QGIS Development Team 2016) from the ASTER Digital Elevation Model version 2 [24], using a 250 m by 250 m resolution for our analyses. Specifically, we considered elevation, a vector of terrain ruggedness [25], and slope angle as potential environmental covariates, each of which might plausibly explain large mammal habitat use patterns in a basin-and-range landscape. Lacking clear information on livestock distribution and space use patterns, we used the Euclidean distance between each camera site and the nearest active herder’s camp as a proxy for the potential impact of human- and livestock-induced disturbance on wildlife space use while acknowledging that Euclidean distance is an imperfect measure of proximity in mountainous landscapes. As free-ranging domestic yaks do not necessarily return to herders’ camps regularly, and we had no other knowledge of their spatial distribution, we omitted any explicit consideration of their role in our models. To account explicitly for the role of vegetation as a potential driver of distributions of herbivores and the carnivores which consume them, we derived an average index of primary productivity from the MODIS/Terra Normalized Difference Vegetation Indices (NDVI) 16-Day L3 Global 250m Grid [26] data product, averaged over the study period. While validation of the relationship between NDVI and living plant biomass was absent for the eastern Pamirs specifically, the relationship has been established in similar neighboring regions of the Qinghai-Tibetan Plateau [27]. Finally, for carnivores, we included two binary variables indicating site-specific documented presence of ibex and argali, respectively. We standardized all covariates prior to analysis and tested for collinearity using Pearson’s correlation coefficient [28]. Correlation among the terrain covariates was high (|*r*| >0.7), and sample sizes were generally low, thus we chose to include only a single terrain covariate in any single model.

For each species, we first developed the best model for detection, *p*, and then investigated candidate models for intensity of use, *ψ*. We considered the static environmental and anthropogenic measures described above as covariates on *ψ*, and standardized occasion date and camera functionality (a binary variable indicating whether a camera was functional for any duration of a sampling occasion), as linear covariates on *p*. For snow leopard, wolf and argali, our model building process first considered models representing a priori hypotheses of linear and quadratic relationships with each of the three terrain covariates and the distance to herder’s camps and a linear relationship to NDVI. For snow leopard and wolf, we hypothesized that in addition to potential patterns of association with terrain covariates, occurrence would correlate positively with the presence of prey, and thus in the first step, we additionally determined which of the two wild ungulate species was better-supported as a covariate on *ψ*, and in the second step, we included that covariate as an additional linear effect with the best models. For argali, we additionally considered the hypothesized linear response to NDVI in combination with the best models identified in the first step, supposing that argali would respond positively to plant growth, while simultaneously selecting other habitat features and making tradeoffs to mitigate predation risk. For rarely detected bear and ibex, we limited our candidate set to single linear covariate models. We used Akaike’s Information Criteria (AIC) to select among candidate models, and considered models for which *Δ*AIC<2 to offer a competitive explanatory value [29].

### Carnivore scat sampling and diet analysis

To assess dietary importance of wild and domestic prey for large carnivores on the reserve (snow leopard, wolf, bear, and domestic dogs) we non-invasively collected scat samples in August 2015 using an opportunistic search-encounter approach during initial camera trap deployment and ungulate surveys, as random or systematic transects were both logistically infeasible and unlikely to yield adequate detection rates. We only collected samples from scats that appeared fresh, a determination based on color, moisture content, and corroborating contextual evidence. Samples for fecal diet analysis were air dried in the field for up to 2 days and stored in sealable plastic bags. For genetic verification of species and individual identification, 3–7 ml of fecal material was gathered from the exterior portion of each scat and stored in silica desiccant at ambient temperatures. While we did make putative distinctions during collection between wild carnivore scats based on macroscopic characteristics, we did not attempt to distinguish even superficially between wolves and domestic dogs. Putative snow leopard scats and putative canid and bear scats were analyzed respectively at Centre for Conservation Genetics at the American Museum of Natural History or the Conservation Biology Lab at the University of Washington to discriminate among carnivore species genetic identity, as we expected our field identification to be subject to substantial error [20]. Genetic species identification was based on carnivore-specific ATP6 region of the mitochondrial genome [30], with subsequent differentiation between wolves and dogs based on 4 single nucleotide polymorphisms in the cytochrome B region developed for this purpose [31].

We randomly selected 20 hairs from each scat sample [15,16,32] to assess the relative frequency of occurrence of various prey items in carnivore diets as estimated by the proportion of scats containing each prey item. We relied on additional scat contents, such as vegetation, bones, or feathers, as necessary to identify other diet items. To identify prey items, we used microscopic morphological features of hairs, such as medullar patterns and cuticular scale patterns, which are distinct for each observed species in the study area [20]. We compared hair against known reference specimens for ibex, argali and marmot collected from the study area, as well as collection materials for livestock and small mammals at the Natural History Museum Vienna (Austria). To account for sampling error [33], we generated 95% bootstrap confidence intervals for the proportion of scats containing each food item [34], simulating 10,000 bootstrap sampling iterations.

Permission to conduct this work was granted by Zorkul Reserve agency and supported by the Institute of Zoology and Parasitology of the Academy of Sciences of Tajikistan. As all research was non-invasive in nature, no animal ethics committee review of our protocols was necessary.

## Results

### Ungulate intensity of use

Of the three wild large carnivores and two wild ungulates present in the Zorkul Reserve during the study period, our camera traps detected argali most frequently, and at the greatest number of sites (Table 1). Under the best-supported model, argali detectability *p*_*ARGALI*_ was positively associated with camera operation, but also negatively associated with date, implying that argali habitat and space use changed over the course of the camera trapping period (Table 2). The best-supported model of argali habitat use intensity *ψ*_*ARGALI*_ included linear effects for NDVI, slope, and distance to herder’s camps (Tables 3 and 4). The relationship between *ψ*_*ARGALI*_ and NDVI was strongly positive, while the association with increasing slope and the distance to herder’s camps changed across the spectrum of observed covariate values. The best model of ibex detection *p*_*IBEX*_ included only a camera operation covariate (Table 2). While we only detected ibex a total of 14 independent times during the observation period and were therefore unable to consider complex covariate effects on ibex use, the best supported model of *ψ*_*IBEX*_ (Table 3) did nonetheless support a positive association between ibex occurrence and distance to the nearest herder’s camps (Table 4).

**Table 1:** Total camera trap detection events by species for the sampling period August 1 to November 8, 2015 in Zorkul Reserve. Independent multiple detection events at the same site within a single sampling occasion were reduced to a single detection for the purposes of occupancy modeling.

Species	Independent Detection Events	Number of Detection Sites
*Snow leopard*	36	14
*Wolf*	32	7
*Bear*	16	11
*Argali*	65	25
*Ibex*	14	6

**Table 2:** Model selection for species specific detection *p* models, where ‘date’ and ‘operate’ respectively indicate camera site and occasion-specific values for the date and camera functionality (on or off). For each species model set, Akaike Information Criterion (AIC) scores consider both model complexity (No. of Parameters) and likelihood. Calculated relative to the lowest scoring or “best” model for each species, *Δ*AIC < 2 indicates the best supported models.

Species	‘unmarked’ Detection Model	No. Parameters	AIC	*ΔAIC*
Snow leopard	~operate	3	161.87	0
	~date + operate	4	163.57	1.7
	(Intercept only)	2	185.79	23.92
	~date	3	187.04	25.17
Wolf	~operate	3	117.07	0
	~date + operate	4	118.27	1.2
	(Intercept only)	2	124.09	7.02
	~date	3	125.99	8.92
Bear	~operate	3	129.93	0
	~date + operate	4	130.16	0.23
	~date	3	130.51	0.58
	(Intercept only)	2	133.06	3.13
Argali	~date + operate	4	288.52	0
	~operate	3	296.2	7.68
	~date	3	324.34	35.82
	(Intercept only)	2	349.55	61.03
Ibex	~operate	3	109.11	0
	~date + operate	4	110.61	1.49
	(Intercept only)	2	117.06	7.94
	~date	3	119.02	9.9

**Table 3:** Model selection for species specific intensity of use *ψ* models, where covariates indicate camera site-specific values of average 250-meter Normalized Difference Vegetation Index (NDVI), slope angle (slope), elevation (elev), terrain ruggedness (rugged), distance to nearest herder’s camp (camp). For snow leopard and wolf models, ‘ibex’ and ‘argali’ covariates were binaries indicating detection/non-detection of those species at a camera site. For each species model set, Akaike Information Criterion (AIC) scores consider both model complexity (No. of Parameters) and likelihood. Calculated relative to the lowest scoring or “best” model for each species, *Δ*AIC < 2 indicates the best supported models.

Species	‘unmarked’ Intensity of Use State Model (*ψ*)	No. Parameters	AIC	*ΔAIC*
Snow leopard	~ slope + ibex	5	156.79	0
	~ slope	4	160.86	4.07
	~ ibex	4	161.24	4.45
	~ (*Intercept Only)*	3	161.87	5.08
	~ slope + I(slope^2)	5	162.81	6.02
	~ rugged	4	163.23	6.44
	~ rugged + I(rugged^2)	5	163.37	6.58
	~ argali	4	163.75	6.96
	~ elev	4	163.77	6.98
	~ camp	4	163.79	7
	~ NDVI	4	163.86	7.07
	~ camp + I(camp^2)	5	164.93	8.14
	~ elev + I(elev^2)	5	165.76	8.97
Wolf	~ NDVI + rugged + I(rugged^2) + camp + I(camp^2)	8	104.04	0
	~ rugged + I(rugged^2)	5	104.86	0.83
	~ rugged + I(rugged^2) + camp + I(camp^2)	7	106.56	2.52
	~ rugged + argali	5	113.96	9.93
	~ slope + I(slope^2)	5	114.99	10.95
	~ rugged	4	115.83	11.79
	~ argali	4	116.08	12.04
	~ (Intercept Only)	3	117.07	13.03
	~ slope + I(slope^2) + camp + I(camp^2)	7	117.72	13.69
	~ slope	4	117.99	13.96
	~ camp	4	118.55	14.51
	~ elev	4	118.86	14.82
	~ ibex	4	118.93	14.89
	~ NDVI	4	119.02	14.98
	~ NDVI + slope + I(slope^2) + camp + I(camp^2)	8	119.35	15.31
	~ elev + I(elev^2)	5	120.04	16.00
	~ camp + I(camp^2)	5	120.39	16.35
	~ elev + I(elev^2) + camp + I(camp^2)	7	122.1	18.06
Bear	~ (Intercept Only)	3	129.93	0
	~ camp	4	130.91	0.98
	~ elev	4	131.66	1.73
	~ rugged	4	131.84	1.9
	~ NDVI	4	131.91	1.97
	~ slope	4	131.93	2
Argali	~ NDVI + slope + I(slope^2) + camp + I(camp^2)	9	272.53	0
	~ slope + I(slope^2) + camp + I(camp^2)	8	278.81	6.28
	~ camp + I(camp^2)	6	280.12	7.59
	~ elev + I(elev^2) + camp + I(camp^2)	8	280.33	7.8
	~ rugged + I(rugged^2) + camp + I(camp^2)	8	281.69	9.15
	~ slope + I(slope^2)	6	286.75	14.22
	~ elev + I(elev^2)	6	287.76	15.23
	~ (Intercept Only)	4	288.52	15.99
	~ NDVI	5	288.8	16.27
	~ camp	5	289.17	16.64
	~ rugged + I(rugged^2)	6	289.84	17.31
	~ slope	5	290.26	17.73
	~ elev	5	290.49	17.96
	~ rugged	5	290.49	17.96
Ibex	~ camp	4	106.1	0
	~ (Intercept Only)	3	109.11	3.01
	~ slope	4	109.94	3.84
	~ rugged	4	110.95	4.85
	~ NDVI	4	111.06	4.96
	~ elev	4	111.09	4.99

**Table 4:** Covariate parameter estimates in the best-supported models of ungulate and carnivore intensity of use. SE indicates Standard Error, z is the z statistic for that parameter, and p(>|z|) is the probability of observing a z score of greater absolute value if the true value of the parameter is zero.

Species	Parameter	Estimate	SE	z	p(>|z|)
*Snow leopard*	*(Intercept)*	1.65	33.74	0.0488	0.9611
	Slope	2.19	1.15	1.8936	0.0583
	Ibex Presence	5.60	73.39	0.0762	0.939
*Wolf*	*(Intercept)*	-2.803	2.188	-1.28	0.2002
	NDVI	-2.390	1.512	-1.581	0.1139
	Camp Distance	-0.608	0.944	-0.644	0.5198
	Camp Distance^2^	-0.687	0.848	-0.810	0.4180
	Ruggedness	-14.803	8.218	-1.801	0.0717
	Ruggedness^2^	-8.652	4.383	-1.974	0.0484
*Bear*	*(Intercept)*	-0.427	0.594	-0.719	0.472
*Argali*	*(Intercept)*	4.992	1.938	2.58	0.01
	NDVI	2.242	1.006	2.23	0.0259
	Slope	0.957	0.826	1.16	0.2468
	Slope^2^	-1.467	0.655	-2.24	0.0252
	Camp Distance	2.200	1.137	1.93	0.0531
	Camp Distance^2^	-3.413	1.523	-2.24	0.0251
*Ibex*	*(Intercept)*	-1.19	0.694	-1.71	0.0868
	Camp Distance	1.19	0.613	1.94	0.0524

### Carnivore intensity of use

Detectability of both snow leopard and wolf was best modeled as dependent on camera operation (Table 2). While the best model of wolf habitat use was complex, incorporating NDVI as well as linear and quadratic effects of the proximity to herder’s camps and terrain ruggedness (Table 4), AIC model selection also supported an alternative model where intensity of use was best modeled by ruggedness alone (*Δ*AIC = 0.83, Table 3). Snow leopard use was best modeled by a positive response to slope and ibex presence (Tables 3 and 4). We only detected bear 16 independent times. As such, covariate effects models of *ψ*_*BEAR*_ were not well-supported in model selection (Table 3). The best model of bear detection *p*_*BEAR*_ included only a camera operation covariate, however, a model incorporating a negative effect of date as well was also competitive (*Δ*AIC = 0.23, Table 2). It is worthwhile to note that in several instances, bears destroyed cameras, leading to a circular relationship wherein camera operation itself was partially dependent upon bear presence or absence.

### Carnivore diet

With the exception of bear scats, we observed considerable discrepancies between the putative and genetically confirmed species identity of carnivore scats (Table 5). Of 151 total samples, we were unable to reliably determine species or adequately amplify DNA from 16 samples, and an additional 9 scats were red fox. Argali and long-tailed marmot were the most frequently detected prey items for all three wild carnivores, although long-tailed marmot was disproportionately prominent in bear diets (Table 6). Wolves and dogs showed the greatest dietary variety. We also detected plant material in the scats of all four carnivores, though never in isolation.

**Table 5:** Frequency of putative and genetically confirmed carnivore scat species identity for scat samples collected August, 2015 in Zorkul Reserve.

	Confirmed Species Identity						
Putative Species	*Snow Leopard*	*Wolf*	*Bear*	*Dog*	*Fox*	*Failed*	Total
*Snow Leopard*	7	-	-	-	4	4	15
*Wolf*	11	75	-	17	5	12	120
*Bear*	-	-	16	-	-	-	16
*Total*	18	75	16	17	9	16	151

**Table 6:** Observed proportion with 95% bootstrapped confidence intervals, of carnivore scats containing each prey item, sampled from scats collected August, 2015 in Zorkul Reserve. Dash “-” indicates no observed occurrence.

Predator	Ibex	Argali	Marmot	Hare	Livestock	Small Mammals	Birds
*Snow leopard (n = 18)*	-	0.61 (0.39–0.84)	0.5 (0.27–0.73)	-	-	0.06 (0–0.16)	-
*Wolf* *(n = 75)*	0.03 (0–0.06)	0.51 (0.39–0.62)	0.56 (0.45–0.67)	0.08 (0.02–0.14)	0.04 (0–0.08)	0.15 (0.06–0.23)	-
*Bear* *(n = 16)*	-	0.06 (0–0.18)	0.94 (0.82–1.00)	-	-	0.06 (0–0.18)	-
*Dogs* *(n = 17)*	0.06 (0–0.17)	0.18 (0–0.36)	0.41 (0.18–0.65)	0.18 (0–0.36)	0.12 (0–0.27)	0.53 (0.24–0.82)	0.06 (0–0.17)

## Discussion

Our findings provide further empirical evidence suggesting that livestock grazing and associated human activities in protected areas have the potential to detrimentally impact large mammals and the relationships among them [3,7,12]. However, at current levels, seasonal grazing in Zorkul Reserve appeared to prompt only some apparent displacement of ungulates, specifically argali, but no perceptible prey shifting from wild to domestic prey by carnivores during the summer months.

Our observations are consistent with a growing body of work showing that pastoral land use in the mountains of Central Asia is more likely to affect the space use of ungulates, especially argali, than that of their predators [3,7]. The humped shape of the association between argali intensity of use and distance to herder’s camps suggests that if argali were indeed responding to, and avoiding, livestock and humans, the effect was only detectable up to some threshold distance. This effect was present even when accounting for other covariates that may influence argali habitat use, implying support for our hypothesis that pastoralism can displace argali. Declining use at the greatest distances may reflect the existence of other herder’s camps that were not included in our estimates of camp proximity. While our modeling suggests a possible shift in ibex space use away from livestock, consistent with previous work [7], the small number of detections was inadequate for meaningful modeling [23]. As others have observed, it is not livestock alone, but rather a combination of livestock, domestic dogs, and human pastoralist activity that negatively affects wild ungulates [9, 12]. Singh et al. [35] found that argali select areas with greater green plant tissue biomass, having observed a seasonal sequence of argali foraging that progressed from graminoids in the spring, to summer forbs (especially *Fabaceae* and *Oxytropis spp*.), and finally semi-shrubs to autumn and winter [36,37]. Our direct observations suggested that livestock in Zorkul Reserve use similar forage, reinforcing our conclusion that livestock displace argali from accessing limited resources [35].

In contrast to the clear signal of the impact of human and livestock presence on argali habitat use, the best model for snow leopards suggested that intensity of use was positively associated with slope, consistent with well-established knowledge of the species’ predilection for steep terrain [38]. The best model also included ibex presence as a nonsignificant predictor of snow leopard occurrence, but given the observed lack of ibex in snow leopard diet, we speculate that this was reflective of overlapping habitat preferences between snow leopard and ibex, rather the importance of ibex as a prey species in the reserve.

Strikingly, the best model of wolf use intensity implied that wolves were actually more likely to occur at sites closer to livestock. However, the large number of parameters in this model relative to the total number of wolf detections calls for cautious, even skeptical interpretation of a possibly overspecified model. Based on our model selection procedure (*Δ*AIC < 2), we could not dismiss a simpler alternative model of wolf use based on terrain ruggedness. In both of the top models for wolves, the humped response of wolf to terrain ruggedness was consistent with patterns of wolf preference for rolling terrain noted by others [39].

Both snow leopard and wolf depend primarily upon wild ungulates [17,20], but our dietary analysis suggests that marmot are also foundational in the seasonal diets of all large carnivores in Zorkul Reserve. While we did not directly address marmot habitat associations, our general observations suggest that marmot, like argali, prefer areas high in green plant biomass, and we note that further research should focus more explicitly on patterns of marmot occurrence in relationship to land use in the region. In much of their range, argali are associated with gentle undulating terrain relative to the overall mountain landscape [40], and are therefore less likely prey for snow leopards than their abundance might otherwise suggest [41]. The observed low ibex abundance and the absence of ibex in sampled snow leopard scats was surprising in light of the dietary importance of ibex and other typically cliff-dwelling ungulates for snow leopards in other regions [40,41]. Two related explanations may account for the unexpected predominance of argali in snow leopard diets in Zorkul Reserve: the disproportionate abundance and availability of argali relative to ibex; and temporal bias in the scats we used to estimate diet. The availability of argali as snow leopard prey may result not only from the greater abundance of argali compared to ibex, but also from argali habitat shifts. For example, argali females typically alter their habitat use for several weeks during the brief lambing period (May-June), moving from open, moderate slopes to steep broken terrain [14], resulting in a short-lived pulse of lambs that may be overrepresented in our estimates of snow leopard prey. Additionally, human and livestock displacement of argali into more rugged terrain [14] may afford snow leopards consistently greater opportunity to prey on argali, functionally increasing the availability of argali as prey for snow leopards. The population level impacts on argali and snow leopards alike under such a scenario would presumably depend on livestock numbers and pasture management. Should livestock numbers or spatial extent increase, the combined effects of competition, displacement, and potentially increased vulnerability to predation may have strongly negative impacts on argali.

Importantly, our study is limited to summer patterns of site use and carnivore diet in a mountainous environment with strong seasonal variation. In winter, argali migrate to winter pastures beyond the boundaries of the reserve, while marmots hibernate, resulting in a very different landscape of potential prey for large carnivores. Furthermore, our estimates of diet and space use were not drawn together from the same sampling period, creating potential confusion in the interpretation of our results. Instead, they represent related, but separate lines of inquiry into the potential impact of livestock on wildlife in Zorkul. Nonetheless, neither livestock numbers, nor the overall distribution of herding activity changed appreciably during the combined study period.

In our results we see complex implications for management of livestock in protected areas that will require pragmatism and careful consideration of local context to achieve conservation goals. The area in and around Zorkul Reserve has been used for pastoral purposes for many generations, and our findings suggest that limited human and livestock presence may be partially compatible with the conservation goals of this particular reserve. However, we also see potential for hidden thresholds in livestock numbers and distribution and therefore emphasize the uncertainty and risks for pasture degradation, human wildlife-conflict, disease transmission, and wild ungulate displacement. Simultaneously, the role of domestic dogs in preventing attacks on livestock should be weighed carefully against their potential negative impacts as predators, competitors, and potential disease vectors. Future management of the Zorkul Reserve should seek to limit grazing, though we suspect that complete elimination would be not only unrealistic but possibly detrimental, as it could foster antipathy towards conservation in local communities. Given the limited capacity for enforcement of wildlife laws, involvement of the local communities who traditionally use the area will be required to affect long-term conservation [42]. Sustainable livelihoods through conservation friendly land-use, community-based wildlife management, and co-management could be powerful tools to replace and reduce the impact and extent of pastoralism, to the benefit of wild ungulates and carnivores [16,43].
